# Hemophagocytic lymphohistiocytosis and histiocytic necrotizing lymphadenitis secondary to hemodialysis catheter-related bloodstream infection caused by Corynebacterium Striatum

**DOI:** 10.1186/s12882-023-03356-2

**Published:** 2023-10-06

**Authors:** ZhiPeng Zhao, Jing Li, Liu Yang, GuangWei Ren, LiHong Zhang, Tao Wang

**Affiliations:** 1 Graduate School of HeBei Medical University, No.361 East ZhongShan Boulevard, ShiJiaZhuang, 050011 China; 2 Department of Nephrology, the First Hospital of HeBei Medical University, No.89 DongGang Road, ShiJiaZhuang, 050030 China; 3 Department of Hematology, the First Hospital of HeBei Medical University, No.89 DongGang Road, ShiJiaZhuang, 050030 China

**Keywords:** Maintenance hemodialysis, Catheter-related bloodstream infection, Hemophagocytic lymphohistiocytosis, Cytokine, Kikuchi disease, Corynebacterium striatum

## Abstract

**Background:**

We herein described the coexistence of hemophagocytic lymphohistiocytosis (HLH) and histiocytic necrotizing lymphadenitis, alternatively known as the Kikuchi disease (KD), secondary to hemodialysis catheter-related bloodstream infection (BSI) caused by Corynebacterium striatum.

**Case presentation:**

A patient on maintenance hemodialysis had developed persistent fever and Corynebacterium striatum was subsequently identified from the culture of both catheter tip and peripheral blood. During mitigation of the BSI, however, his fever was unabated and ensuing workup further found thrombocytopenia, hyperferritinemia, hypertriglyceridemia, low NK cell activity and a surge in serum CD25 levels. Moreover, biopsy of the bone marrow and lymph node detected histopathological evidence of hemophagocytosis and KD, respectively. Upon these abnormalities, the title-bound diagnosis was considered and the patient was eventually recovered from the treatment of dexamethasone instead of antibiotics. Consistently, aberrations in his serum CD25 levels and NK cell activity had subsided two months after discharge.

**Conclusions:**

Arguably, this encounter offered a unique chance to unravel the principal pathogenic cascade in immunobiology that made the three entities one disease continuum. As such, our work may add new understandings of HLH and/or KD secondary to severe infections in general and excessive release of cytokines in particular among patients with kidney diseases. The resultant early diagnosis is crucial to initiate appropriate treatment and improve the survival of patients with these challenging and potentially life-threatening disorders.

## Background

Hemophagocytic lymphohistiocytosis (HLH) is a rare and potentially fatal yet under-recognized disease. It is essentially a hyperinflammatory state caused by dysregulation in natural killer (NK) cells and/or cytotoxic T lymphocytes, resulting in activation and proliferation of macrophages and lymphocytes with uncontrolled cytokine overproduction and hemophagocytosis [1]. Reportedly, secondary HLH is usually associated with malignancies, autoimmune diseases or infections [2]. Meanwhile, uncontrolled activation of cytotoxic T lymphocytes may also lead to histiocytic necrotizing lymphadenitis, alternatively known as Kikuchi disease (KD) [3]. Under more rare circumstance, HLH and KD can occur concurrently and yield clinical outcome different from HLH alone [4]. Nevertheless, predominant cause of the above said activation differs in each country, which suggests a specific genetic background or differences in suspected triggering factors, particularly infections [1].

We herein described the coexistence of HLH and KD during an episode of hemodialysis catheter-related bloodstream infection (BSI) caused by Corynebacterium (C.) striatum. Arguably, this encounter may offer a unique chance to unravel the principal pathogenic cascade in immunobiology that made the three entities one disease continuum. Starting with the nature of C. striatum, HLH was focused with clinical manifestations, followed by participation of serum CD25 and features of KD, and wrapped up with its relevance in the latest pandemic. In the last context, Soy et al. in their recent work on HLH ‘inspired’ by the COVID-19 pandemic called for increased rheumatologists’ awareness of the life-threatening rare complication of HLH to prevent a possible misdiagnosis in the presence of the viral infection [5]. Of importance, maintenance hemodialysis (MHD) patients with COVID-19 also had a high fatality due to systemic hyperinflammatory cytokine release [6]. Although the concept of hyperinflammation or cytokine storms in COVID-19 is now widely accepted, however, it has been cautioned that there still remain multiple unknowns [7]. Nevertheless, our work may greatly facilitate expeditious recognition of HLH and/or KD over severe infections among patients with kidney diseases.

## Case report

**Fig. 1:**
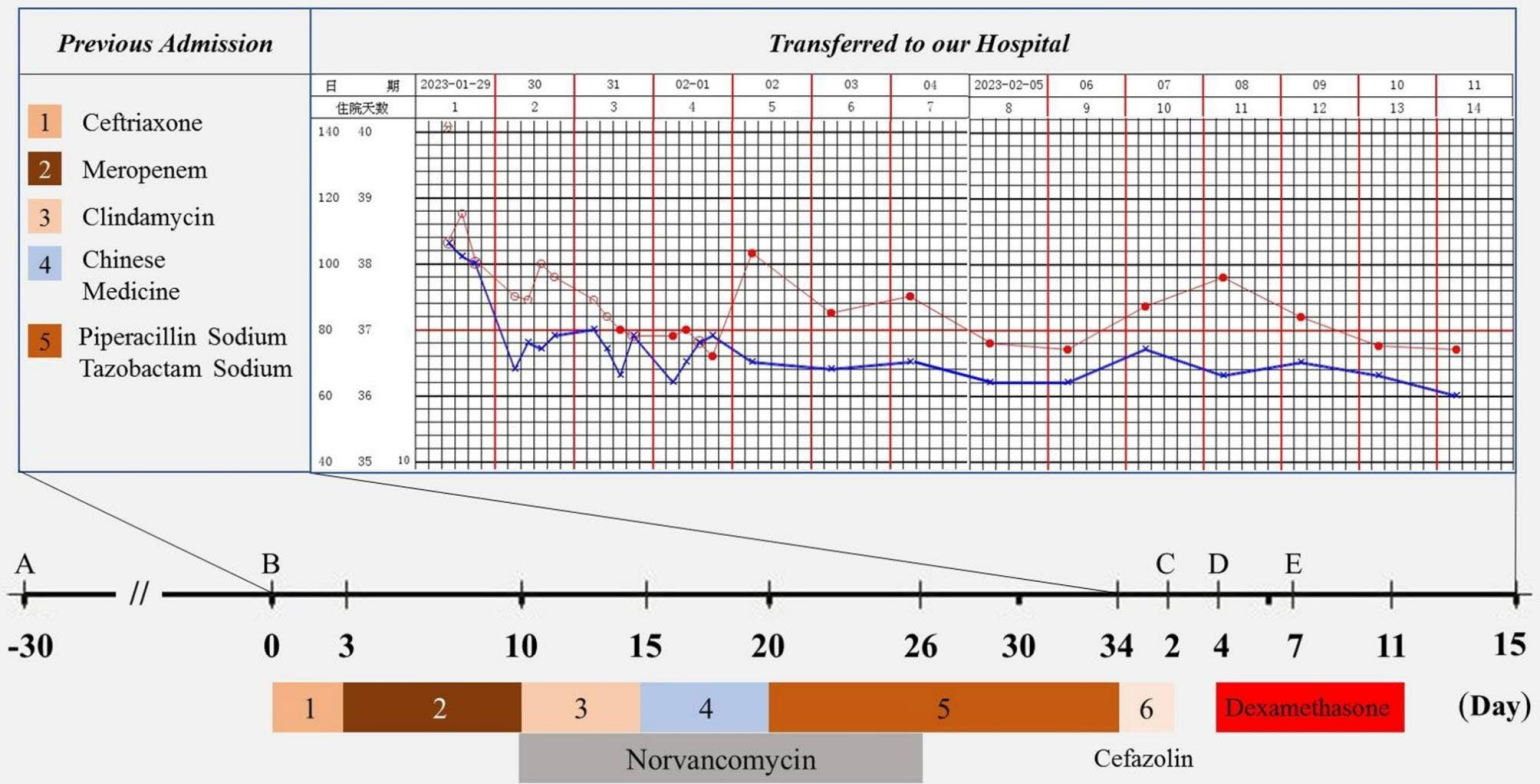
Managements of the patient prior to and after his transfer to us. Red circle and blue cross denote body temperature and pulse, respectively. The open circles indicate continous monitoring of the vital signs, during which fever was contained by lysine acetylsalicylate. **A**: the initiation of hemodialysis using femoral catheter. **B**: onset of fever and replacement of the femoral catheter with a new one to the internal jugular vein. **C, D, E**: biopsy of the lymph nodes and bone marrow (BM) aspiration, detection of hemophagocytosis in BM and determination of serum CD25 level/NK cell activity, respectively

A 56-year-old diabetic man on MHD was referred to us for persistent fever. Until then, the patient had started MHD through a right femoral vein catheter two months ago and was again admitted to the same local hospital for fever one month later. His temperature fluctuated between 38.0-39.5ºC with the highest readout accompanied by rigor during the dialysis session. Laboratory tests showed leukocyte 2.83 × 10^9^/L with 69.7% neutrophil, hemoglobin 91 g/L, platelet 124 × 10^9^/L, serum creatinine (Scr) 1242.0 µmol/L, procalcitonin (PCT) 5.97 ng/mL (reference, < 0.05ng/mL), ferritin 409 ng/mL (25-350ng/mL), fibrinogen 4.17 g/L (2.0-4.0 g/L). Treatment was begun with Ceftriaxone and Meropenem, and accordingly changed over to Norvancomycin when microbial culture of the peripheral blood and tip of the removed catheter both grew C. striatum. Despite remission of the infectious indices, however, his fever remained unabated. Finally, the patient was transferred to us after a hospital stay of 34 days (Fig. 1).

**Fig. 2:**
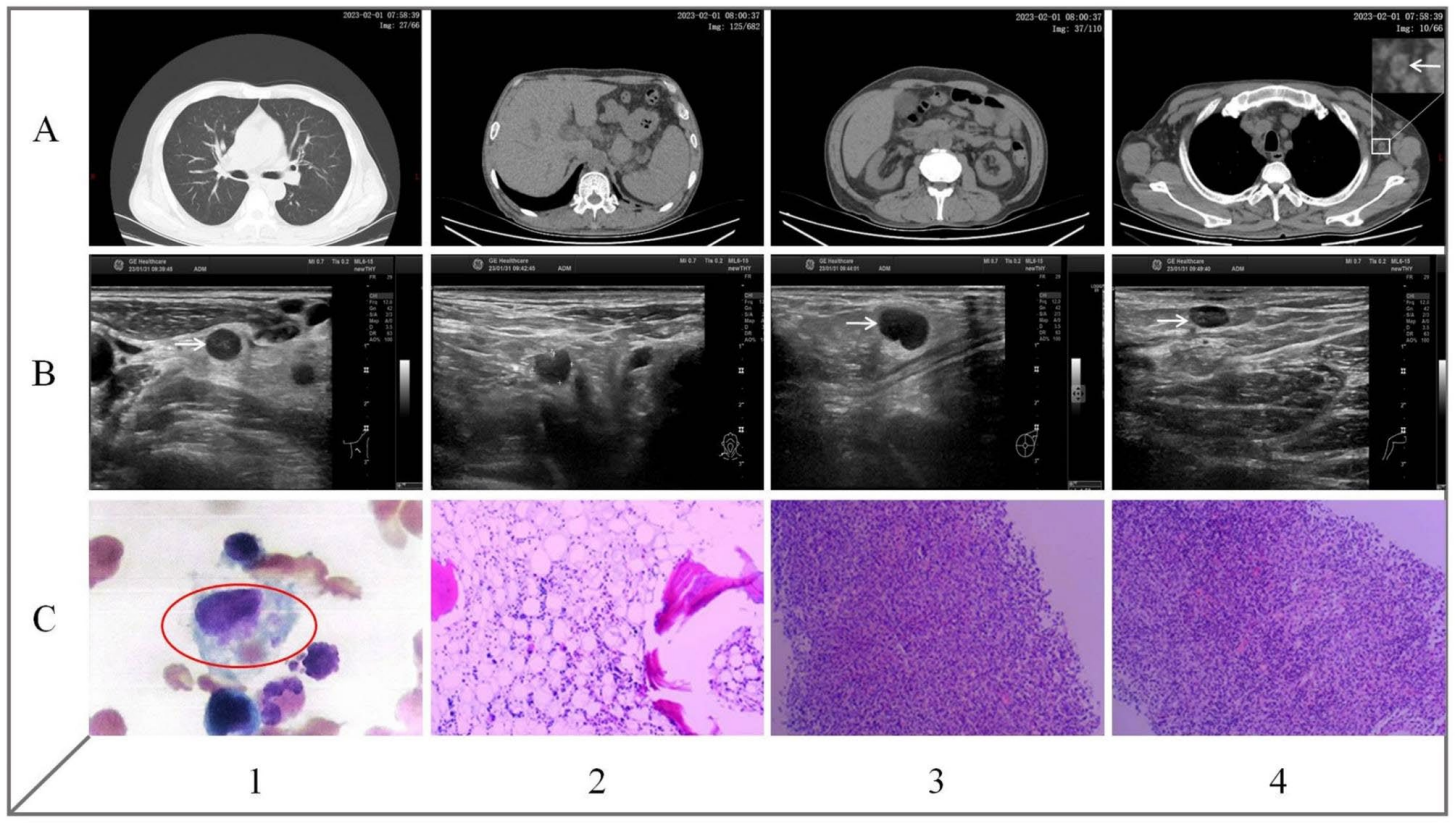
Imaging and morphologic findings of the index patient. **A1-A3**: Pulmonary, abdominal and pelvic CT scan shows no sign of infection, absence of hepatosplenomegaly and kidneys with slightly diminished size. A4: Mediastinal window depicts enlarged left axillary lymph node, with the ring-shape nodal necrosis (arrow). **B1-B4**: US image obtained at the same time as the CT scan shows a somewhat matted, hypoechoic nodal mass at the cervical, supraclavicular, axillary and inguinal sites, with an irregular perinodal border (arrow), suggesting perinodal infiltrates. **C1-C2**: Bone marrow aspiration and biopsy reveal trilineage hematopoiesis with hemophagocytosis (red circle). **C3-C4**: Lymph node biopsy finds that left cervical and axillary lymph nodes were filled with CD3^+^CD8^+^ T-lymphocytes surrounded by histiocytes in the necrotizing lesions, which was consistent with a diagnosis of KD.

On arrival, the patient was febrile, pale and asthenic with right jugular vein catheter visible upon a temperature of 38.6 ºC and BP 140/85 mmHg. Systemic examination revealed enlarged superficial lymph nodes with tenderness, normal auscultation of lungs and no hepatosplenomegaly. Initial workup showed anemia, thrombocytopenia, elevated C-reaction protein, PCT, lactate dehydrogenase, triglycerides and serum ferritin (Table 1). By comparison, all tumor markers, immunological and microbiological tests yielded no remarkable finding. Specifically, serological tests excluded as follows: hepatitis B and C, cytomegalovirus, Epstein-Barr virus, respiratory syncytial virus, parainfluenza virus, adenovirus, parvovirus B19, enterovirus, herpesvirus, Rickettsia, mycoplasma, chlamydia, Brucella spp., toxoplasmosis, fungi and mycobacterium tuberculosis. Metagenome next generation sequencing and repeated cultures of his blood were also negative. Moreover, there was no sign of cerebral, thoracic and abdominal infection on CT scans, whereas enlarged lymph nodes were confirmed by ultrasound at the cervical (the largest one 15.7 × 7.8 mm), supraclavicular (7.3 × 5.1 mm), axillary (15.8 × 8.2 mm) and inguinal (7.8 × 3.6 mm) areas. Subsequently, bone marrow aspirate and biopsy found active trilineage hematopoiesis with scattered hemophagocytic macrophages. In addition, needle biopsies of the left cervical/axillary lymph nodes identified histiocytic necrotizing lymphadenitis. Upon these findings (Fig. 2), further investigation detected a surge in soluble CD25 level and lower NK cell activity. Taken together, a diagnosis of HLH and KD secondary to the catheter‑related BSI was considered. After a hematology consult, the patient was treated with daily intravenous dexamethasone of 5 mg for one week and eventually discharged with a normal temperature. Of note, his relevant clinical indices showed clear improvements two months after discharge.

**Table 1:** Chronological results of the laboratory tests

	Admission	Discharge	Follow-up	Reference Range
Leukocytes (×10^9^/L)	8.9	3.9	3.0	3.5–9.5
Neutrophils (%)	95.3	56.5	55.1	40.0–75.0
Lymphocytes (×10^9^/L)	0.25	0.90	0.67	1.10–3.20
Hemoglobin (g/L)	84	91	109	130–170
Platelets (×10^9^/L)	70	122	207	125–350
sCD25 level (pg/mL)	27,888	NA	7655	< 6400
NK cell activity (%)	4.83	NA	10.5	> 15.1
CRP (mg/L)	120.8	12.7	4.0	< 5.0
Precalcitonin (ng/mL)	1.55	0.67	0.31	< 0.05
Albumin (g/L)	30.9	27.8	37.0	40.0–55.0
AST (U/L)	54.8	26.6	14.5	15.0–40.0
ALT (U/L)	14.8	7.7	10.9	9.0–50.0
LDH (U/L)	663.0	357.0	343.2	120.0-250.0
ALP (U/L)	88.0	67.0	100.0	45.0-125.0
Bilirubin (µmol/L)	9.5	9.6	13.1	< 23.0
BUN (mmol/L)	22.0	20.3	20.3	3.1-8.0
Scr (µmol/L)	742.5	872.6	649.9	57.0–97.0
Cholesterol (mmol/L)	2.62	4.05	4.30	2.80–5.17
Triglycerides (mmol/L)	3.02	2.54	0.75	0.20–1.70
HDL-cholesterol (mmol/L)	0.70	0.82	1.31	1.03–1.55
LDL-cholesterol (mmol/L)	1.48	2.74	2.60	< 3.37
Ferritin (ng/mL)	2312.0	553.1	182.9	23.9-336.2
Fibrinogen (g/L)	3.72	3.43	4.11	2.38–4.98
PT (s)	9.7	11.0	12.2	9.4–12.5
APTT (s)	16.0	26.5	23.7	25.1–36.5

All results were pre-dialysis ones and the follow-up values were determined at two months after discharge. CRP: C-reactive protein; AST: aspartate aminotransferase; ALT: alanine aminotransferase; LDH: lactate dehydrogenase; ALP: alkaline phosphatase; BUN: blood urea nitrogen; Scr: serum creatinine; PT: prothrombin time; APTT: activated partial thromboplastin time.

## Discussion

To the best of our knowledge, this is the first work causally and coherently addressing the C. striatum infection and the ensuing HLH/KD in the clinical setting of hemodialysis. These three entities were discussed with a focus on the serum CD25 and converged on cytokines abnormality in MHD patients. In fact, reports of HLH in MHD patients with infection remain comparative paucity and there was only one letter briefly described a fatal case during *Pseudomonas* septicemia in similar setting [8]. Hence, relevance of these less familiar yet no less deadly entities was better highlighted in our field of specialty.

Catheter-related BSI is common in MHD patients. In this regard, Corynebacterium striatum is increasingly associated with human invasive infections worldwide [9], especially in MHD patients with a central venous catheter [10]. By origin, Corynebacteria are Gram-positive, catalase-positive, aerobic or facultative anaerobic, generally non-motile rods [9]. By nature, it is part of microbiota of skin and nasal mucosa of humans and has been progressively reported as the second most prevalent coryneform microorganism in immunocompromised patients. By virulence, C. striatum is prone to evolve into antimicrobial resistance in hospital environment. By pathogenicity, they have an important nature of promoting pro-inflammatory behavior in macrophages by direct microbe-macrophage interaction [11]. Indeed, such a nature was supposedly responsible for human necrotizing lymphadenitis due to C. pseudotuberculosis [12].

Secondary HLH has a wide range of causes, symptoms, and outcomes. Nevertheless, it essentially arises from a defective granule (perforin/granzyme)-mediated cytotoxicity in the NK cells and cytotoxic T lymphocytes, which impairs or abolishes their function in the killing of target cells and activated T lymphocytes [2]. Consequentially, this deficiency results in persistent activation of lymphocytes and histiocytes (macrophages) that forms the lymphohistiocytic infiltrates found in the organs of patients with HLH, as well as hypersecretion of inflammatory cytokines. Accordingly, HLH is characterized by fever, peripheral blood cytopenia in at least two cell lineages, splenomegaly, increased levels of serum ferritin, hypertriglyceridemia and/or hypofibrinogenemia, hemophagocytosis, low or absent NK cell activity and increased levels of CD25 [13]. The diagnosis of HLH can be established if five out of these eight criteria are met, whereas our patient was only short of the organomegaly. The diagnosis, however, should be made with caution since sepsis can be a presenting feature of BSI and there are considerable clinical and laboratory overlaps between sepsis and HLH.

Better known as soluble interleukin-2 receptor α chain, CD25 is extensively involved in immunity or self-tolerance [14]. Extremely high level of serum CD25 has been recognized as a hallmark of HLH [15], however, it is also seen in the T-cell lymphoma [16]. The latter possibility has been carefully contemplated and needle biopsy of the enlarged lymph nodes from two different areas found no evidence of such a malignancy and hemophagocytosis. Comparing with the excisional biopsy, this approach traded detection accuracy for safety since our patient with thrombocytopenia required low molecular weight heparin for dialysis. Besides, observations at the follow-up provided extra support as change of the serum CD25 levels was in line with previously reported patterns in the HLH [13]. Although increased levels of serum CD25 are not disease specific, in combination with a persistent preceding infection and histopathological evidence of hemophagocytosis, it may reasonably add to the diagnostic certainty of HLH secondary to severe hemodialysis catheter-related BSI.

Kikuchi disease is a self-limiting disorder of unknown origin characterized by painful cervical lymphadenopathy with prolonged fever [9]. Generally, the cervical lymph nodes are most commonly affected, but virtually any nodal chain can be involved with none exceeding 2.5 cm in the largest dimension [4]. On histopathology, the affected lymph nodes usually show patchy necrotic areas with karyorrhexis surrounded by activated histiocytes [17]. These enlarged lymph nodes were easily noticeable on ultrasound as hypoechoic nodal mass with an irregular perinodal border. On CT scans, most of them had perinodal infiltrates that may end up with central necrosis. Apparently, our current findings are consistent with these abnormalities (Fig. 2a4) and we are more inclined to consider KD instead of an epiphenomenon related to secondary HLH. There are only 30 reports of HLH accompanied by KD nowadays and this combination is predisposed to occur in children with a female to male ratio of 2:1 [17]. Interestingly, hemophagocytosis in these patients is only identified in the bone marrow but not in the peripheral lymph nodes [4]. Furthermore, cervical lymph nodes in patients with HLH alone have demonstrated homogeneously enhancing nodes without perinodal infiltrates or central necrosis, which can be seen in KD alone or in KD associated with HLH [18]. As such, it is possible that HLH can be accompanied by KD, and vice versa, since both diseases are associated with marked activation of lymphocytes and histiocytes.

The cardinal principle for treatment of HLH requires a triple simultaneous approach: supportive measures against life-threatening presentations, concomitant control of the triggering factors (mainly infection) and suppression of inflammatory response [1]. Dexamethasone remains the cornerstone to suppress hypercytokinemia, especially in secondary HLH [19]. Prognostically, HLH is one of the most critical clinical disorders in adults with a mortality rate of 41-57% [20]. By comparison, patients with HLH accompanying KD have a relatively good outcome [4].

Likewise, our MHD patient also manifested a rapid remission. It is likely due to the facts that patients on MHD have impaired cellular immune function and hemodialysis procedure per se helps to remove the cytokines from the blood [21]. Nonetheless, COVID-19 is known to render systemic hyperinflammatory cytokine release thus high fatality in MHD patients [6]. We speculated that MHD may attenuate but not abrogate the aberrant inflammatory response, and such an attenuation is insufficient to offset a surge in cytokines during a severe viral infection and/or the uremic state may even potentiate the detrimental effects of the elevated cytokines, at least in a subgroup of MHD patients. Consistently, renal impairment is a major risk factor for the increased mortality in COVID-19 patients [22]. Inspired by the latest pandemic, our work on the HLH/KD induced by infection and mediated by cytokines intends to help nephrologists be more prepared for these unexpected challenges, which may in turn support the otherwise vulnerable MHD patients weathering over them.

## Conclusion

From the approach of nephrology, we shared our experience in the management of HLH secondary to hemodialysis catheter-associated bloodstream infection. Undoubtedly, early and accurate diagnosis of severe BSI, HLH or both is essential to initiate appropriate treatment and improve the survival of MHD patients with these life-threatening disorders.

## Data Availability

De-identified source data and material would be provided by the corresponding author upon request.

## Abbreviations

BSI: bloodstream infection
C.: Corynebacterium
HLH: hemophagocytic lymphohistiocytosis
KD: Kikuchi disease
MHD: maintenance hemodialysis
NK cell: natural killer cell
Scr: serum creatinine
